# Rare nasosinusal tumors: Case series and literature review

**DOI:** 10.1016/S1808-8694(15)31106-X

**Published:** 2015-10-19

**Authors:** Roberta de Paula Araújo, Érika Ferreira Gomes, Dalgimar Beserra de Menezes, Lidiane Maria de Brito Macedo Ferreira, Adson Sales do Nascimento Rios

**Affiliations:** 1 ENT Resident - HGF SESA/SUS; 2 ENT Residency Program Preceptor - Hospital Geral de Fortaleza, Otorhinolaryngologist; 3 MD. Pathologist. Assistant Professor of General Pathology - Federal University of Ceará Medical School; 4 ENT Resident - HGF SESA/SUS; 5 ENT Resident - HGF SESA/SUS. Hospital Geral de Fortaleza - SESA/SUS

**Keywords:** nasal cavity, neoplasm

## Abstract

Tumors of the nasal cavity and paranasal sinuses are unusual pathologies found in clinical practice. Approximately 0.8% of all human cancers are located in this area. Despite being rare, nasosinusal neoplasms usually manifest through nonspecific symptoms that are common to numerous inflammatory pathologies. The aim of this study is to describe a series of rare nasosinusal tumors, including esthesioneuroblastomas, central giant cell granulomas, extramedullary plasmocytomas, nasosinusal hemangiopericytomas, neurofibromas and cemento-ossifying fibromas, diagnosed at the Fortaleza General Hospital. We, hereby, briefly review each of the aforementioned pathologies, stressing the need for a precise histological diagnosis for proper treatment in each case.

## INTRODUCTION

Nasosinusal malignant tumors are rare, representing less than 3% of head and neck cancers and 0.8% of all human cancers^1^. Approximately 55% originate in the maxillary sinuses, 35% in the nasal cavity, 9% in the ethmoid and 1% in the frontal and sphenoid sinuses2. In the USA, the incidence of nasal cavity tumors is less than 1 case in 100 thousand persons per year^2^. With the exception of non-epithelial tumors, nasosinusal cancer is a disease that affects adults, being more frequent in men above 50 years of age^2^.

Tumors in this region usually cause unspecific and common symptoms, including inflammatory diseases. Nasal obstruction (61.2%), epistaxis (40.8%), facial pain (39.2%) and local infection (23.9%) are the most commonly reported initial symptoms^1^. Such fact, associated with the low incidence of these tumors and, often times, the difficulties associated with histopathology, contribute to delays in diagnosis and treatment.

## CLINICAL CASES

### Case 1

ATS, female, 16 years, coming from Acarape/CE, came to us complaining of epistaxis followed by right side nasal obstruction for 3 years. Anterior rhinoscopy showed a hyperemic tumoral lesion, brittle, involving the upper portion of the right nasal cavity (RNC), extending all the way to the middle meatus.

**Figure 1:**
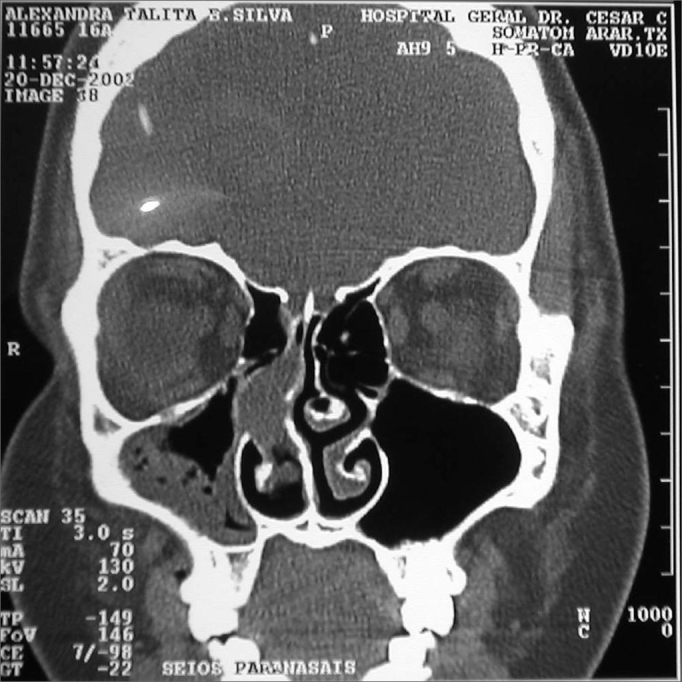
CT scan. Coronal view of a Stesioneuroblastoma (case 1).

Nasal cavity and paranasal sinuses CT scan showed a dense polypoid mass, well defined, in the right nasal cavity, fully obliterating the middle meatus and the sphenoethmoidal recess, with middle turbinate and poorly defined ipsilateral posterior ethmoidal air cells. We could also see a thickening of the right maxillary and sphenoidal sinuses mucosa (Figure 1).

Incision biopsy followed by nasal packing was carried out in the outpatient ward. Pathology exams showed clusters of small cells of round hyperchromatic nuclei amidst fibril stroma, matching signs of stesioneuroblastoma. Having a diagnosis of Kadish B (nasal cavity and paranasal sinuses involvement), we proposed a mid-facial degloving resection, followed by radiotherapy (RxT). Still during the preoperative, the patient had intensive epistaxis, requiring arteriography and selective right maxillary artery catheterization for tumor embolization.

**Figure 2:**
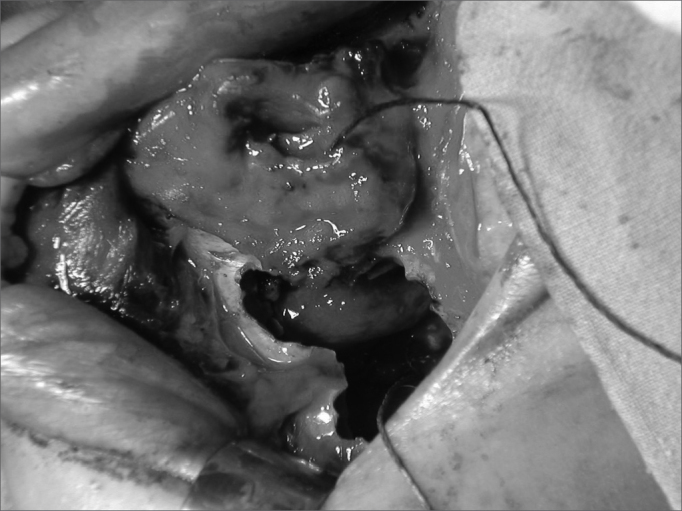
Middle-face degloving with anterior maxillary sinusotomy, showing stesioneuroblastoma (case 1).

In the eighth post-embolization day, we carried out a cranio-facial tumor resection. The lesion occupied the upper portion of the right nasal cavity, measuring approximately 4cmx3cmx1.5cm. Anterior maxillary sinusectomy showed no tumoral mass in the right maxillary sinus (Figure 2). The medial wall of the maxillary sinus was resected and the ethmoid lamina cribosa was drilled all the way to the dura-mater, to guarantee surgical mass removal. The patient was then referred to RxT, and today is in the 8th month of follow up, without signs of recurrence.

### Case 2

**Figure 3:**
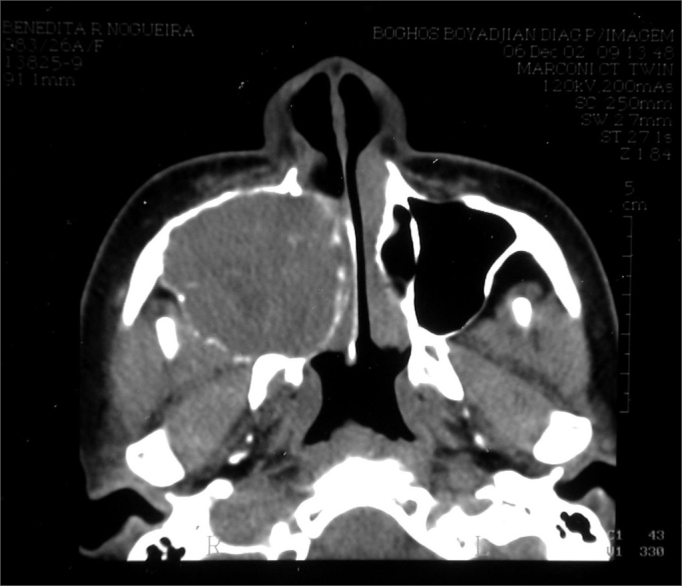
CT scan - axial cross section of a GCCG (case 2).

BRN, Female, 26 years, came from Fortaleza/CE, came to us complaining of a bulging on the hemiface and right nasal obstruction, with 8 months of development. She reported she had a tooth removed (1st right pre-molar) one year before. Her CT scan showed an expansive solid large heterogeneous mass occupying her right maxillary sinus, with expansion and small areas of cortical invasion. The lesion extended to the hard palate and ipsilateral tooth alveolar process, to the RNC and partially obstructed the inferior orbital fissure (Figure 3).

**Figure 4:**
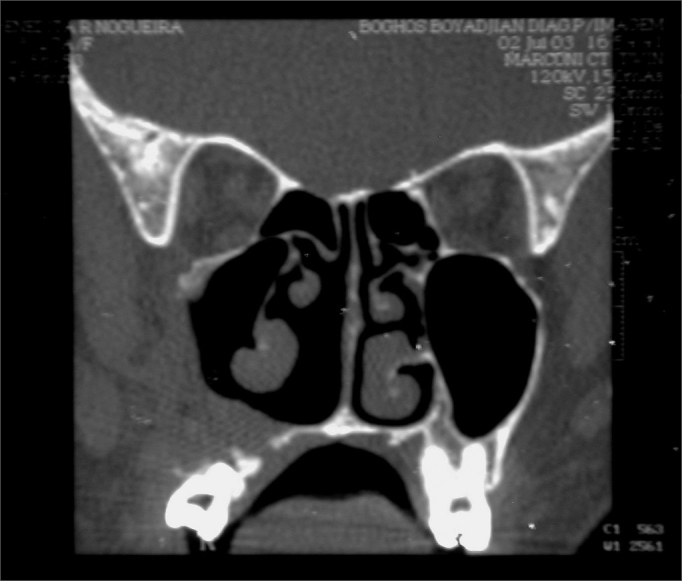
CT scan - coronal view of a GCCG post operative (case 2).

When we biopsied her through a sublabial antrostomy, we could see no anterior bone wall on the right maxillary sinus and a brittle encapsulated bulky and vascularized tumor. An AP study showed tumor sections reproducing giant multinucleated cells inserted in the fibrous stroma of variable density, with ovoid and fusiform cells proliferation, in a clinical picture matching that of giant cells central granuloma (GCCG). Treatment proposed was direct maxillectomy through mid-facial degloving. The patient is in her 24th month of follow up, with no evidences of tumor recurrence (Figure 4).

### Case 3

**Figure 5:**
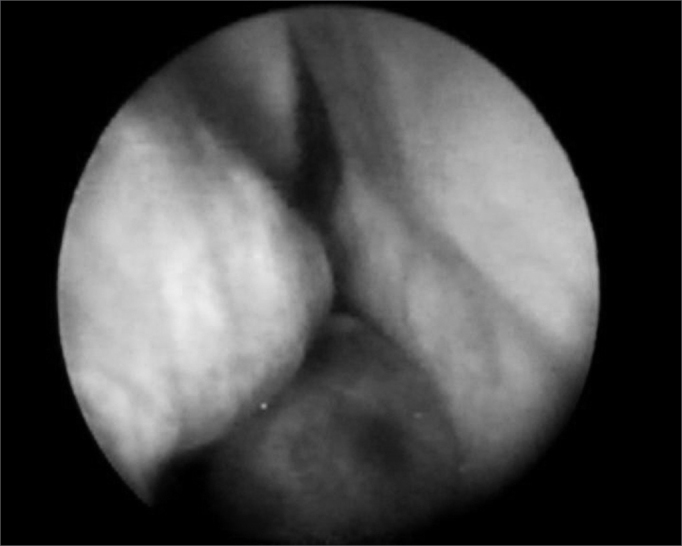
RNC Extramedullary Plasmocytoma - nasal fibroscopic view. (case 3).

**Figure 6:**
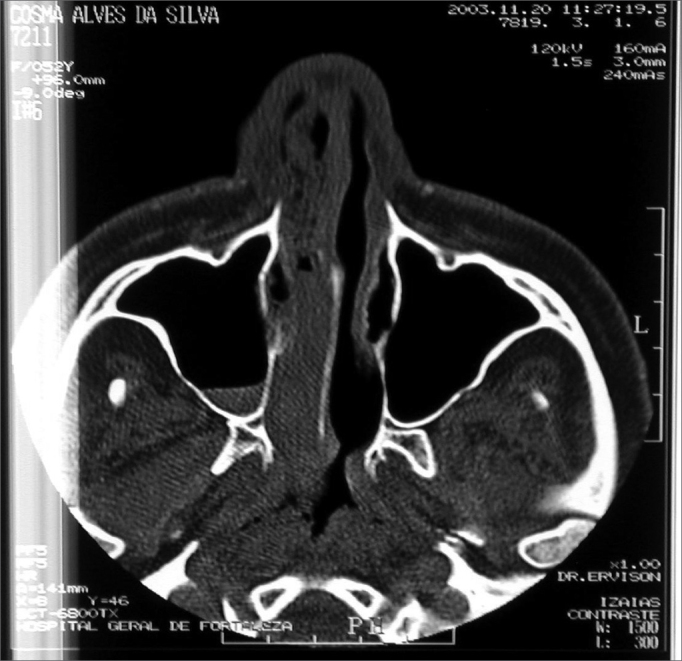
CT scan - axial view of Extramedullary Plasmocytoma (case 3).

CAS, female, 52 years, coming from Fortaleza/CE, complained of epistaxis for 3 years, followed by right side nasal obstruction. Her otorhinolaryngological physical exam was normal. Through nasal-endoscopy we could see a tumor on the RNC floor, extending to the nasopharynx (Figure 5). Paranasal sinuses CT scan showed an expansive homogeneous mass, of soft tissue density in her right nasal cavity and nasopharynx, without evidence of bone erosion (Figure 6). We carried out an incisional biopsy, showing connective and vascular neoformation, with intense infiltration of plasmocytic cells and respiratory-type cell, matching signs of plasmocitic granuloma. We ordered lab exams in order to rule out systemic disease (multiple myeloma). Blood tests, bone marrow aspirate and biopsy, together with protein electrophoresis were all within normal ranges. The tumor was excised by endoscopy, with no evidences of bone invasion or that of adjacent structures. The patient was referred to complementary RxT, and is in her 8th month of follow up, without evidences of tumor recurrence or systemic disease.

### Case 4

**Figure 7:**
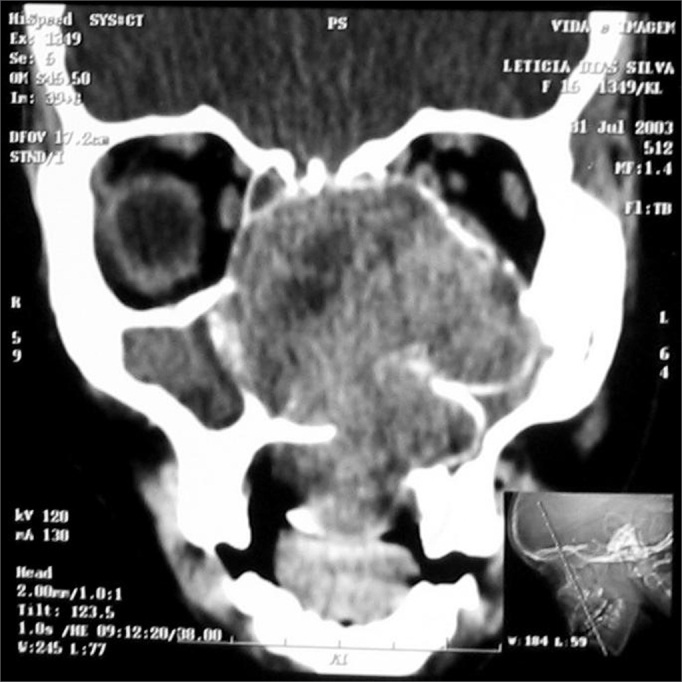
CT scan - coronal view of a nasosinusal hemangiopericytoma (case 4).

LDS, 15 years, female, coming from Fortaleza/CE, came to us complaining of tooth ache and bolus lesion on her palate to the left, followed by epistaxis, hemifacial bulging and ipsilateral exophthalmus, of about one year of evolution. Paranasal sinuses CT scan showed an expansive mass in the left nasal cavity, extending anteriorly to the orbit apexes and supra-sellar region, destroying the pterygoid lamina. The lesion expanded to the ipsilateral sphenoidal and maxillary sinuses and to the hard palate, uptaking little contrast. (Figure 7).

At our first biopsy attempt, there was an important bleeding, then we chose to do an arteriography and tumor embolization, which presented expressive regression. We did an excision biopsy through anterior maxillary antrostomy, and we only saw the tumoral capsule and the necrotic tissue in a large cavity within the maxillary sinus.

AP study showed connective tissue with mixed infiltrate of inflammatory cells and vascular clefts with collarette of cells with round nuclei; areas of mucinous material build up and deposits of hemosiderin pigment. We concluded it was an angiomatoid tumor, with no signs of malignancy, with a differential diagnosis between glomangioma and nasosinusal hemangiopericytoma. Tumoral cells laying around vascular spaces that showed only vimentin in the immunohistochemistry exam, negative for actin, smooth muscle actin, CD31 and CD34, is more compatible with nasosinusal hemangiopericytoma.

**Figure 8:**
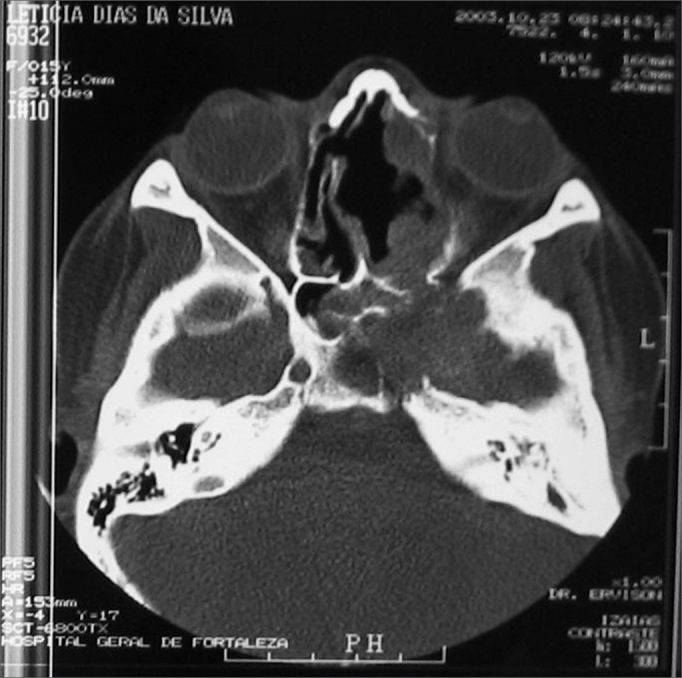
CT scan - axial view of a relapsed nasosinusal hemangiopericytoma (case 4).

Three months after the surgical procedure, the patient returned complaining of epistaxis. During nasal-endoscopy we noticed a residual tumor in her RNC floor, extending to the soft palate. CT scan showed a lesion measuring about 5cm × 3cm, with bone destruction and rarefaction, involving the orbital floor, lateral wall and maxillary sinus floor. The lesion was destroying the clivus, ethmoid and sphenoid sinuses on the left side, invading the parasellar region and involving the cavernous sinus (Figure 8).

The patient underwent RxT and chemotherapy (QT), and died about one year after the diagnosis, with clinical signs of intracranial hemorrhage.

### Case 5

**Figure 9:**
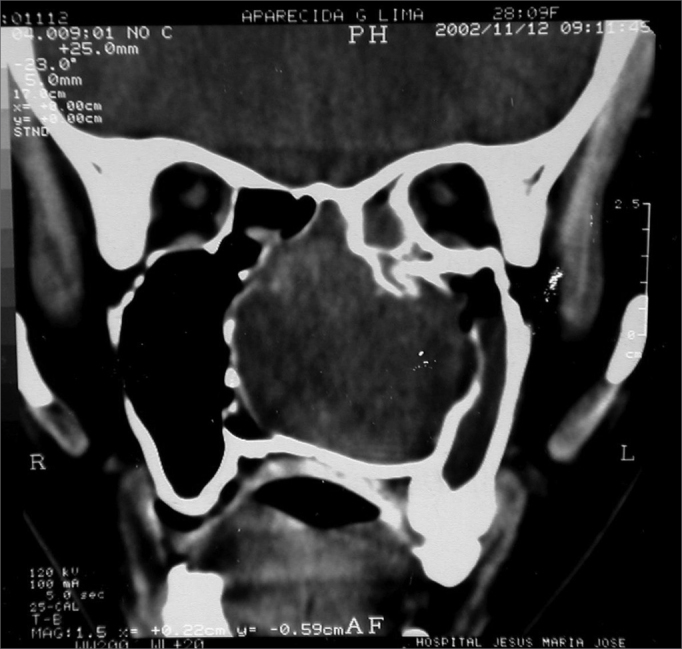
CT scan - coronal view of a neurofibroma (case 5).

AAGL, female, 23 years old, coming from Maranguape/Ce, with previous history of epistaxis and left side nasal obstruction for 5 years. She had a hyperemic and non-bleeding tumor in her LNC, and ipsilateral bulging on the nose, maxillary and palate regions. Her CT scan showed an expansive lesion in her left nasal cavity (LNC), occupying the rhinopharynx and ipsilateral maxillary and ethmoidal sinuses, with right side nasal septum deviation and no signs of bone lysis (Figure 9).

She underwent an incisional biopsy, which showed proliferation of elongated and fusiform cells, some with nuclei arranged in bundles, matching signs of neurofibroma. We carried out a sublabial maxillary antrostomy to resect the lesion, which measured approximately 9cmx9cmx2cm. She is being followed up for 17 months now, and there is no evidence of tumor relapse.

### Case 6

**Figure 10:**
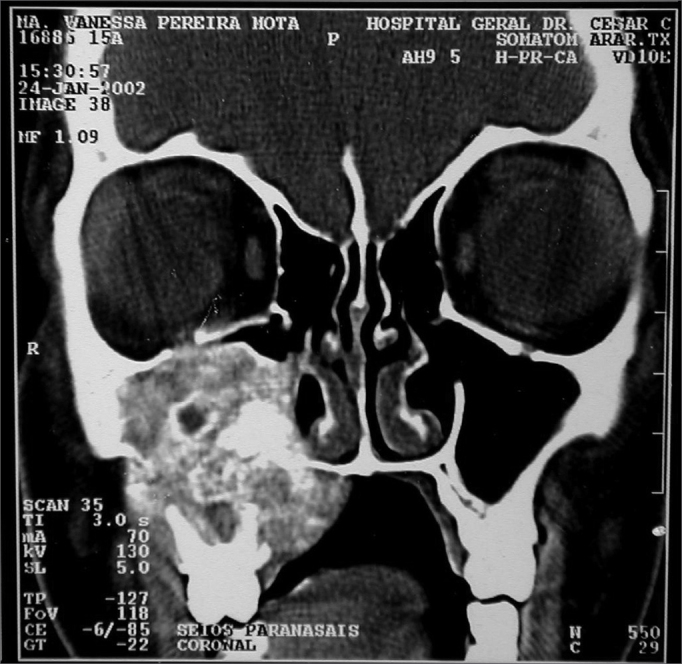
CT scan - coronal view of a cement-ossifying fibroma (case 6).

MVPM, female, 15 years old, had a bulging in her right infraorbital region, periodontal ulceration of the upper molars and submucosal bulging of her ipsilateral hard palate that had been evolving from one year. She did not have epistaxis or nasal obstruction. CT scan showed an expansive lesion with concentric calcifications in her right-side maxillary sinus, destroying its walls and invading the hard palate (Figure 10).

We carried out an incisional biopsy through anterior maxillary antrostomy, and we saw a sponge-like tumor. The AP study showed partialy immature bone tissue, and within its trabeculae there was fibrous connective tissue, with no evidence of significant atypia, leading us to consider it a cement-ossifying fibroma. We proposed surgical excision, but the patient refused it.

## DISCUSSION

### Stesioneuroblastoma

The stesioneuroblastoma is a neurogenic tumor that originates from the olfactory region neuroepithelium^3^. It corresponds to 6% of the malignant nasal and paranasal tumors; and to 0.3% of the upper airway and digestive tract cancers^4^. It happens at any age, with similar prevalence in both genders^5^. It was initially described by Berger et al. in 1924, under the term “esthésioneuroépithéliome olfactif”^5^. Between 1924 and 1989, less than 300 cases were reported5. This figure almost reached 1,000 after a literature review carried out by Broich et al., and most of them were diagnosed after 1997, probably because of histological diagnosis difficulty in small-cell tumors and restricted access to immunohistochemistry^5^.

Symptoms are unspecific and common to most tumors and benign diseases of the nasal cavity - unilateral nasal obstruction and epistaxis are the most frequent ones^5^. Headache, visual disorders and hyposmia are also reported^3^^,^^5^. Kadish's classification divides the tumors in three stages: A (tumors restricted to the nasal cavity), B (tumors involving the nasal cavity and paranasal sinuses) and C (tumors extending to beyond the paranasal sinuses -orbit, skull base or metastasis)^5^.

CT scan is the radiologic exam of choice, which shows a homogeneous mass in the nasal cavity, of soft tissue density and uniform and moderate contrast uptake^6^. It is important to asses a possible erosion of the lamina papyracea, lamina cribosa and ethmoidal fovea. MRI is useful to determine intraorbital or intracranial tumor extension^6^.

The treatment proposed for the case is the one advocated by most authors: cranio-facial resection followed by radiotherapy^3^^,^^6^^,^^7^. A metanalysis carried out in 2001 correlated the survival rate in five years with treatment modality - 65% with surgery and radiotherapy, 51% with radiotherapy and chemotherapy, 48% surgery alone, 47% with surgery plus radiotherapy and chemotherapy and 37% with radiotherapy alone^7^.

Neck lymphadenopathy is an important prognostic factor, and the survival rate is 64% in patients without neck metastasis and 29% in patients with metastasis^7^. Local recurrence happens in 15 to 20% of the cases6, which has been reported even after 10 years of initial treatment, requiring prolongued treatment, especially in young patients. Distant metastasis after local lesion control is relatively rare (4 to 8%)^5^.

### Giant Cells Central Granuloma (GCCG)

GCCG was only recognized as a clinical entity after 1953, and was first described by Jaffe who called it giant cells central “repair” granuloma^9^. It is a rare pathology, corresponding to less than 7% of the benign maxilla and mandible tumors^10^. More than 70% of the cases affect the mandible, anteriorly to the 1st molar^9^, and the mandible/maxilla ratio is of 2–3:1. There are reports of GCCG, however, in the temporal bone, paranasal sinuses, skull base and orbit, among others^10^. More than 60% of the cases happen in patients below 30 years of age, with predominance for females^11^.

The pathogenesis of GCCGs is still little understood, and there are controversies regarding its neoplastic origin or reactional^12^. Recent studies show proliferative GCCG activity, suggesting a deregulation in the cell cycle that can contribute to the disease's pathogeny. Alterations in the expression of cyclin D1, a protein that regulates the cell cycle, were found in GCCGs and in giant cell tumors (GCT)^12^. Similar immunohistochemistry patterns for other proteins that regulate the cell cycle (MDM2 and p53) were also observed in GCTs and GCCGs^12^. Having this in mind, some authors consider these diseases a continuation of the same disease^12^^,^^13^.

According to clinical behavior, the GCCGs are classified in aggressive and non-aggressive forms^11^. The former has a slow growth, usually asymptomatic, with no invasion of the bone cortical and low recurrence likelihood^11^. Now, the aggressive form has fast and painful growth, with cortical invasion and tendency to recurr^11^. No histological or immunohistochemical characteristic has proven to have any correlation with tumor behavior^11^^,^^14^.

Histologically we see CD68+ multinucleated giant cells and mononuclear cells in a fibrous stroma^12^^,^^14^. The GCCG does not have pathognomonic radiologic alterations, and it can simulate other bony lesions^10^. The majority (87.5%) present as a radiolucent expansive lesion, uni or multilocular, with well outlined borders (56%) or not so well defined borders (30%)^11^. More aggressive maxilla lesions may invade the maxillary sinus floor, the orbit and the nasal cavity^11^. One may find mandible cortical bone rupture, teeth shifting or tooth root resorption^11^.

Treatment is based on en-bloc surgical resection or resection by curettage, depending on the clinical and radiologic aspect of the lesion^9^^,^^11^. Since it is a more aggressive lesion, we chose en-bloc resection in this case, and we attained a very satisfactory result. One study showed a 50% recurrence rate after curettage, compared to only 10% after complete lesion resection^10^. The most used alternative treatment modalities are: steroid injection; calcitonin injection or intranasal spray; and alpha-2a interferon injection^9^.

### Extramedullary Plasmocytoma

The plasmocytoma is a rare neoplasia originated from B cells. It may present as three variants: multiple myeloma (MM), osseous solitary plasmocytoma and extramedullary plasmocytoma (EMP)^15^^,^^16^. MM, initially described in 1846^16^, is the most common and spread form of plasmatic cells tumor. The osseous solitary plasmocytoma is characterized by trabeculated and multicystic intra-osseous lesions, usually involving the spine, pelvis and femur^16^.

EMPs represent about 4% of the non-epithelial nasosinusal tumors and about 0.4% of the head and neck tumors^16^. It develops in the submucosal tissues of the upper airways in 80% of the cases, with decreasing incidence in the nose, paranasal sinuses, nasopharynx, palatine tonsil, thyroid, gums and hypopharynx^15^. It prevails in males at the rate of 3–4:1, and it is more frequent in those patients above 40 years of age^17^.

The most common symptoms of nasal cavity EMP, as seen in our case, are progressive nasal obstruction and intermittent epistaxis^15^^,^^17^. Pain is uncommon, unless there is secondary infection or bone destruction^16^. Physical or endoscopic exams of the nasal cavity reveal a submucosal, polypoid and lobulated tumor18, usually not ulcerated and brittle^15^. The anterior rhinoscopy of this case did not show tumoral lesion, calling for the need of complementary diagnostic approaches.

The CT scan shows an expansive mass, usually homogeneous, with minimum to no bone erosion. Light microscopy shows groups of plasmocytic cells with different degrees of differentiation, varying from mature and small cells to immature and multinucleated^16^, associated to a rearrangement of adjacent structures.

Laboratorial exams must be ordered in order to rule out MM: CBC, protein electrophoresis, bone marrow aspirate and biopsy, electrolytes, radiological studies of the skeleton, among others. Some patients, between 5% and 32%, will develop systemic manifestations of MM after some years, which require prolongued follow up19. Treatment is based on surgery and complementary radiotherapy. EMPs are radiosensitive and regional control can be achieved in more than 80% of the cases^17^^,^^19^.

### Nasosinusal Hemangiopericytoma (NSHP)

The NSHP is a rare mesenchymal tumor of the nasosinusal region, characterized by a pattern of prominent perivascular growth^20^. Since its initial description in 1942, the definition of this disease as a specific clinical entity has been questioned^21^. Despite the terminology, it is believed that this disease represents a neoplasia that is clinically and pathologically different from the conventional soft tissue hemangiopericytoma (STHP)^20^^,^^21^. Some authors associate it with the glomus tumor, considering it a variant of the former or even a hybrid between the glomus tumor and the STHP^20^, due to the similarities in biological behavior, cytological profile and immunophenotypes.

Between 15% and 30% of all STHP happen in the neck and head region, they account for less than 1% of vascular neoplasias^21^^,^^22^. The NSHP affects preferentially those patients in the sixth and seventh decades of life in both genders^20^^,^^21^. The most common clinical picture is unilateral nasal obstruction and/or epistaxis, with a polypoid mass in the nasal cavity^20^^,^^21^. Radiologic exams reveal opacification caused by polypoid mass, sometimes associated to bone erosion and sclerosis ^21^.

Histologically, the NSHP is a submucosal, non-capsulated lesion, with diffuse growth pattern and prominent perivascular hyalinization^20^^,^^21^. It has a myoid appearance without cellular pleomorphism, with tumoral cells organized in sheets or fascicles^20^^,^^21^. They bear no specific immunohistochemical marker. There is frequent expression of vimentin, actin and smooth muscle actin^20^^,^^21^. Contrary to the STHP, it does not manifest CD34^20^^,^^21^. In the case hereby presented, the tumoral cells manifested only vimentin.

In general, NSHPs are very resistant. They manifest aggressive behavior as in the case hereby presented, they are uncommon and there are reports in the literature of lesions with local destructive potential and metastasis^21^. Histological characteristics that may be associated with aggressive behavior include a larger number of mitotic figures, increase in cellularity with cell pleomorphism and necrosis^21^. Large lesions (larger than or equal to 6.5cm) and incomplete first resection are all associated with a worse prognosis^21^.

Treatment is surgical, varying from simple polypectomy to resection with broad free margins^20^^,^^21^. Local recurrence happens in 17% to 40%^21^ of the cases, probably due to residual tumor. Prognosis is favorable, with 5-year-disease-free survival rate of 74.2% and in 10 years it is 64.4%^21^. Radio and chemotherapy can be used for metastatic disease and residual or non-resectable primary tumors^21^.

### Neurofibroma

Benign neural tumors, represent about 45% of the head and neck tumors^23^. The neurofibroma, a neoplasia originated from the Schwann cells, is the most common type, affecting most frequently the skin^23^. About 25% to 45% of neurofibromas happen in the head and neck, and only 4% involve the nasal cavity and paranasal sinuses^24^.

It manifests as isolate lesion or multiple tumors, as it happens to the Type 1 neurofibromatosis (NF1) or Von Recklinghausen's disease^23^. Almost all patients with NF1 will develop a neurofibroma at some point in their lives^25^. The potential for malignization is of 2.6%, and in the syndrome it varies between 3% and 15%^24^.

Despite being non-capsulated, these are well-outlined lesions. Light microscopy shows a proliferation of all the elements of the peripheral nerve, spread in a loose and disorganized pattern in a myxoid stroma. There is a predominance of Schwann cells with fusiform nuclei^23^. This disarranged architecture helps differentiate it from schwannomas. Treatment is surgical^24^^,^^25^.

### Cement-Ossifying Fibroma - COF

The COF is a rare, non-odontogenic mesodermic tumor^26^^,^^27^. According to the classification from the World Health Organization, it is considered a variant of the cementifying fibroma, which represents a subgroup of cementomas^27^. The cementomas, fibrous-osseous lesion with cement, are divided in four subgroups, according to their clinical, histopathological and radiological characteristics: benign cementoma, cementifying fibroma, cement periapical dysplasia and gigantiform cementoma^26^^,^^28^.

With still controversial etiopathogenesis, it is believed that these lesions stem from the periodontal membrane, which contains blast cells capable of forming bone, fibrous tissue and cement^26^. Tooth extraction or traumas have been considered possible causal factors^26^.

The majority occur in the molar and pre-molar regions of the mandible^29^. They may also involve the maxilla and, more rarely, other paranasal sinuses. Some studies show their predilection for females, more frequent in the 4th and 5th decades of life^29^. They usually manifest as an asymptomatic mass, well outlined and slow growth, diagnosed in a routine radiologic exam^29^. It may be associated with local pain, teeth loss or dental occlusion alterations^29^. Large maxillary lesions may cause facial asymmetry, rhinorrhea, proptosis and diplopia^29^. There are reports of aggressive tumors, especially when located in the maxilla and ethmoid^26^.

Radiologically, they manifest as a well outlined mass, with soft tissue density and foci of ossification, there may be bone destruction^30^. Light microscopy shows fibrous tissue with spheroid bony lamellar trabeculae, permeating a fibrous stroma in close association with fibroblasts. Masses of acellular basophile material are shown as concentric lines, identified as cement^30^.

Treatment is surgical, and there are reports of remission after incomplete resection^26^^,^^29^. In principle, radiotherapy is contra-indicated, because of tumor resistance and the possibility for malignization^29^^,^^30^.

## CONCLUSION

Non-epithelial tumors of the nasal cavity and paranasal sinuses are rare pathologies, with extremely varied etiopathology, clinical behavior, treatment and prognosis. The lack of symptom specificity usually delays diagnosis. Having all of this in mind, the physician must be attentive to the diverse number of diseases that may affect this region, and histopathology is extremely necessary for properly approaching these cases.
